# A retrospective analysis of maternal complications and newborn outcomes of general anesthesia for cesarean delivery in a single tertiary hospital in China

**DOI:** 10.1186/s12871-022-01753-y

**Published:** 2022-07-06

**Authors:** Yang Bao, Ting Zhang, Ling Li, Changqing Zhou, Minxian Liang, Jie Zhou, Chunling Wang

**Affiliations:** 1 Department of Anesthesiology, Qilu Hospital of Shangdong University, 107 Wenhua Xi Road, Jinan, 250012 Shandong China; 2 Department of Anesthesiology, Critical Care and Pain Medicine, Beth Israel Deaconess Medical Center, 330 Brookline Avenue, Boston, MA 02215 USA; 3 UMass Memorial Hospital University of Massachusetts Chan Medical School, 119 Belmont St, Worcester, MA 01605 USA

**Keywords:** General anesthesia, Neuraxial anesthesia, Morbidly adherent placenta, Cesarean delivery

## Abstract

**Background:**

Either neuraxial anesthesia or general anesthesia can be performed for cesarean delivery. Generally, neuraxial anesthesia is the first choice with the risk and benefit balance for both the mother and fetus. However, general anesthesia is also applicable most commonly in the emergent setting. This study analyzed maternal complications associated with general anesthesia for cesarean delivery and suggested lowering pregnancy-related maternal and newborn adverse outcomes.

**Methods:**

With the approval of the Institutional Ethics Review Board (No: 2017016), data on cesarean delivery and related anesthesia were collected from the Electronic Health Record System from 1/1/2013 to 12/31/2016. Statistical software STATA version 15.1 was used for data analyses. All statistical tests were two-sided, and a level significance of 0.05 was assumed.

**Results:**

The rate of general anesthesia for cesarean delivery increased steadily during 2013–2016, 3.71% in 2013 to 10.23% in 2016 (*p* < 0.001). Repeat cesarean delivery among general anesthesia group increased significantly from 16.22% in 2013 to 54.14% in 2016 (*p* < 0.001). Morbidly adherent placenta (MAP) was the first reason among pregnancy-related complications, which accounted for 33% in total in general anesthesia group (38% in 2013 to 44% in 2016). The laryngeal mask airway (LMA) was used in airway management, and the proportion of LMA increased from 28.38% in 2013 to 92.99% in 2016 (*p* < 0.001). There were significant differences in newborn outcomes between general anesthesia and neuraxial anesthesia groups, including newborn weight, newborn Apgar score at 1 min and 5 min and newborn admission to the NICU (*p* < 0.001).

**Conclusions:**

The growing incidence of general anesthesia was consistent with the trend of rising repeat cesarean delivery and MAP. low newborn Apgar score and high newborn admission to the NICU in general anesthesia group compared with neuraxial anesthesia group. The LMA was performed safely for airway management with enough fasting and careful gastric volume evaluation.

## Background

Cesarean delivery (CD) rates vary tremendously in different countries and regions [1, 2]. To reduce the cesarean delivery rate, guidelines on trials of labor after cesarean delivery (TOLAC) have been developed and updated in the United States and some European countries [3]. However, the trend of cesarean delivery is still on the rise often due to the increase of maternal age [4].

Cesarean delivery not only potentially increases the risk of intraoperative complications but also causes maternal complications for future gestation, including adhesions of the uterus, morbidly adherent placenta (MAP) (placenta previa, accreta, increta, percreta) and uterine rupture [5, 6]. The high rate of placenta previa associated with uterine scarring and the high incidence of MAP make anesthesia management for cesarean delivery challenging [7]. General anesthesia (GA) or conversion from regional to general anesthesia was chosen passively to deal with massive hemorrhage and latent coagulation disorder.

This study aimed to identify causes associated with maternal complications to choose general anesthesia for cesarean delivery, and to make suggestions for lowering maternal and newborn adverse outcomes related to subsequent cesarean delivery.

## Methods

With approval from Qilu Hospital of Shandong University Research Ethics Committee (No: 2017016), we collected cesarean delivery and related anesthesia data in the Electronic Health Record System from 1/1/2013 to 12/31/2016. Maternal demographic characteristics and complications were included, and newborns’ physical characteristics and health status were documented. All cesarean delivery patients with general anesthesia were recruited from 2013 to 2016, and patients undergoing cesarean delivery with neuraxial anesthesia were matched at a ratio of 3:1 according to maternal age and delivery year. General anesthesia techniques were divided into two subgroups according to airway management with laryngeal masks and endotracheal tubes.

### Statistical analysis

Continuous variables were summarized with mean values and standard deviations, and categorical variables were summarized with frequencies and percentages. The distribution of categorical data was analyzed by Pearson’s chi-squared test or Fisher’s exact test, and the difference in continuous variables was compared by two-sample t test, Wilcoxon rank-sum test, one-way analysis of variance or Kruskal–Wallis test when appropriate. We investigated anesthesia methods and general anesthesia techniques over the years by the Cochran–Armitage trend test. The odds ratios (ORs) with 95% confidence intervals (CIs) of anesthesia methods and newborn babies’ Apgar score status for potential factors were estimated by unconditional logistic regression.All statistical analyses were carried out with STATA version 15.1 (Stata Corporation, College Station, TX, USA). All reported probabilities (*p* value) were two-sided, and less than 0.05 was considered statistically significant.

## Results

### Maternal characteristics and obstetric complications and newborn outcomes of parturients accepted cesarean delivery with general anesthesia during 2013–2016 (Table 1)

**Table 1:** Maternal characteristics and obstetric complications and newborn outcomes of parturients who accepted cesarean delivery with general anesthesia during 2013–2016

Variables	2013 (*N* = 74)	2014 (*N* = 200)	2015 (*N* = 173)	2016 (*N* = 314)	*P* value^#^
**Maternal Age(y)**	30.0 ± 5.9	29.4 ± 4.9	30.2 ± 5.8	30.9 ± 4.8	0.004
**Stratified Age (y)**					
18–28	31(41.89)	95(47.5)	76(43.93)	97(30.89)	< 0.001
29–33	19(25.68)	64(32)	45(26.01)	131(41.72)	
34–49	21(28.38)	41(20.5)	52(30.06)	86(27.39)	
Missing	3(4.05)	0(0.0)	0(0.0)	0(0.0)	
**BMI(kg/m2)**	28.86 ± 4.85	28.47 ± 4.18	28.14 ± 4.51	28.73 ± 4.07	0.729
**BMI group**					
< 25.5	13(17.57)	34(17)	34(19.65)	48(15.29)	0.567
25.5–28	14(18.92)	53(26.5)	43(24.86)	93(29.62)	
28–31	18(24.32)	58(29)	41(23.7)	66(21.02)	
> = 31	17(22.97)	41(20.5)	37(21.39)	75(23.89)	
Missing	12(16.22)	14(7)	18(10.4)	32(10.19)	
**Gestational age (Days)**	256.9 ± 20.3	249.1 ± 24.8	246.8 ± 23.3	248.9 ± 24.5	0.012
**Stratified Gestational age (Days)**					
175–259	35(47.3)	120(60)	111(64.16)	183(58.28)	0.072
260–270	16(21.62)	44(22)	41(23.7)	81(25.8)	
271–277	13(17.57)	23(11.5)	11(6.36)	29(9.24)	
278–301	10(13.51)	13(6.5)	10(5.78)	20(6.37)	
Missing	0(0)	0(0)	0(0)	1(0.32)	
**Categorization of Maternal complications**					
No	0(0)	0(0)	0(0)	1(0.32)	0.002
Neurological disease and/or mental disorders	8(10.81)	5(2.5)	7(4.05)	4(1.27)	
Coagulation deficiency	21(28.38)	57(28.5)	26(15.03)	64(20.38)	
Pregnancy-related Complications	39(52.7)	122(61)	128(73.99)	231(73.57)	
Autoimmune disease	4(5.41)	9(4.5)	7(4.05)	10(3.18)	
Others	2(2.7)	7(3.5)	5(2.89)	4(1.27)	
**Emergency or Elective**					
Emergency	46(62.16)	103(51.5)	145(83.82)	264(84.08)	< 0.001
Selective	24(32.43)	95(47.5)	26(15.03)	40(12.74)	
Missing	4(5.41)	2(1)	2(1.16)	10(3.18)	
**Primary or Repeat**					
Primary	58(78.38)	128(64)	96(55.49)	139(44.27)	< 0.001
Repeat	12(16.22)	71(35.5)	76(43.93)	170(54.14)	
Missing	4(5.41)	1(0.5)	1(0.58)	5(1.59)	
**Twins**					
No	70(94.59)	185(92.5)	169(97.69)	303(96.5)	0.071
Yes	4(5.41)	15(7.5)	4(2.31)	11(3.5)	
**Anesthesia technique**					
LMA	21(28.38)	146(73)	145(83.82)	292(92.99)	< 0.001
ETT	53(71.62)	54(27)	27(15.61)	22(7.01)	
Missing	0(0)	0(0)	1(0.58)	0(0)	
**Newborn Outcomes**					
Newborn weight(g)	2672.2 ± 756.0	2652.9 ± 825.4	2605.5 ± 828.9	2672.9 ± 832.1	0.816
**Apgar Score at 1 min**					
0–4	5(6.76)	11(5.5)	12(6.94)	21(6.69)	0.928
5–7	10(13.51)	18(9)	16(9.25)	36(11.46)	
8–10	58(78.38)	161(80.5)	138(79.77)	249(79.3)	
Death	1(1.35)	10(5)	7(4.05)	8(2.55)	
**Apgar Score at 5 min**					
0–4	0(0)	4(2)	3(1.73)	8(2.55)	0.764
5–7	6(8.11)	12(6)	9(5.2)	15(4.78)	
8–10	67(90.54)	174(87)	154(89.02)	283(90.13)	
Death	1(1.35)	10(5)	7(4.05)	8(2.55)	
**NICU**					
No	57(77.03)	142(71)	124(71.68)	174(55.41)	< 0.001
Yes	16(21.62)	47(23.5)	39(22.54)	130(41.4)	
Missing	1(1.35)	11(5.5)	10(5.78)	10(3.18)	
**Total Hospital Days**	10.9 ± 7.2	11.9 ± 9.6	14.4 ± 12.3	10.8 ± 8.1	< 0.001
**Hospital Days after Delivery**	7.6 ± 3.7	6.6 ± 5.2	7.3 ± 4.5	5.7 ± 3.2	< 0.001

*Indications: BMI* Body mass index, *LMA* Laryngeal mask airway, *ETT* Endotracheal tube, *NICU* Neonatal intensive care unit

^#^*P* values were derived using chis-squared test for each categorical variable and Wilcoxon rank sum test for continuous variables, after excluding the corresponding missing value

Maternal age was approximately thirty years old, and the increase in maternal age can be seen during 2013–2016, with a significant difference in maternal age distribution between these four years (*p* = 0.004). There were no differences in maternal BMI at delivery among the GA groups during 2013–2016.

There were significant differences in maternal complication distribution in the GA group from 2013 to 2016, *p* = 0.002. The proportion of cases with pregnancy-related complications to total general anesthesia cases increased from 52.7% in 2013 to 73.99% in 2015 and 73.57% in 2016.

There were also great changes between cesarean delivery characteristics in the general anesthesia group. Although the number of both primary and repeat cesarean deliveries increased, the rate of repeat cesarean delivery increased significantly from 16.22% in 2013 to 54.14% in 2016, *p* < 0.001.

LMA used for airway management was 28.38% in 2013, with a steep growth to the highest of 92.99% in 2016. The increasing trend of LMA used in the GA group presented great differences from 2013 to 2016, *p* < 0.001 (also in Fig. 2).

The gestational age in the GA group was lower than that in the neuraxial anesthesia (NA) group. Preterm birth was common, with an average gestational age below 37 weeks (equal to 259 days) in the GA group. Total hospital days and hospital days after operation in the GA group were longer than those in the NA group.

### The rate of cesarean delivery from general anesthesia to total cesarean delivery each year during 2013–2016 (Fig. 1)

**Fig. 1:**
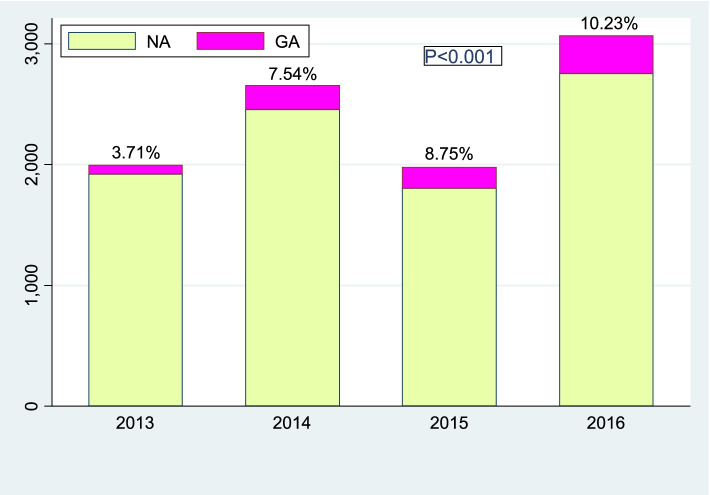
The rate of GA in all CDs each year during 2013–2016. Indications: NA, neuraxial anesthesia; GA, general anesthesia; CD cesarean delivery

The rate of general anesthesia in all cesarean delivery was 3.71% in 2013, steadily trending up to 10.23% in 2016, while the number of cesarean deliveries also increased during 2013–2016, except for slight fluctuations in 2014. There were significant differences in the rate of general anesthesia during 2013–2016, *p* < 0.001.

### General anesthesia with LMA and ETT was chosen for the rate of cesarean delivery each year during 2013–2016 (Fig 2)

**Fig. 2:**
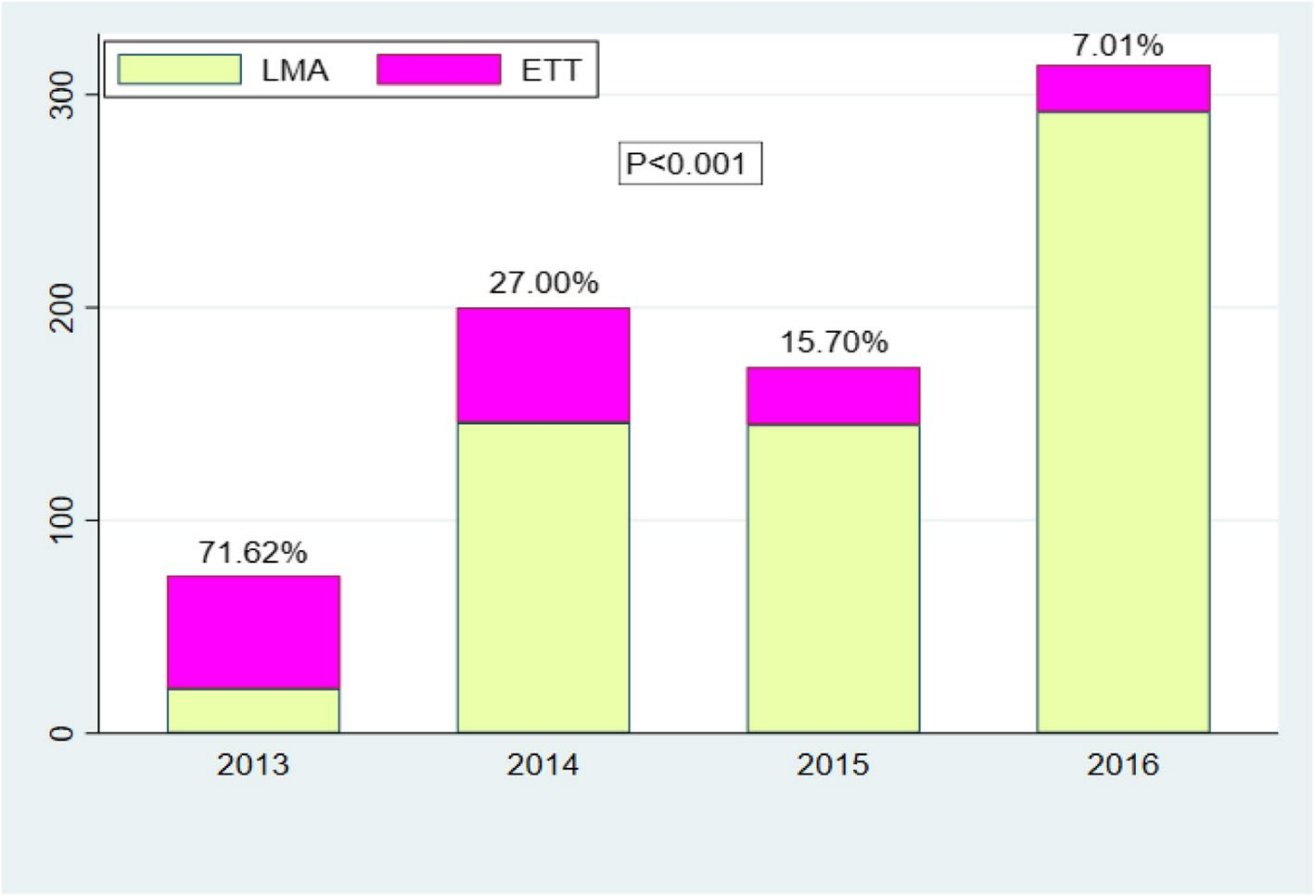
The rate of CD choosing GA with LMA to CD with ETT each year during 2013-2016. Indications: CD, cesarean delivery; GA, general anesthesia; LMA, laryngeal mask airway; ETT, endotracheal tube

LMA used in airway management accounted for 28.38% (21/74) in 2013, while LMA accounted for 92.99% (292/314) in 2016, with a rapid increase year by year. There were great differences between LMA and endotracheal tube (ETT) for general anesthesia airway management during 2013–2016, *p* < 0.001.

### The chart of maternal complications and pregnancy-related complications in the GA group yearly from 2013 to 2016 (Fig. 3)

**Fig. 3:**
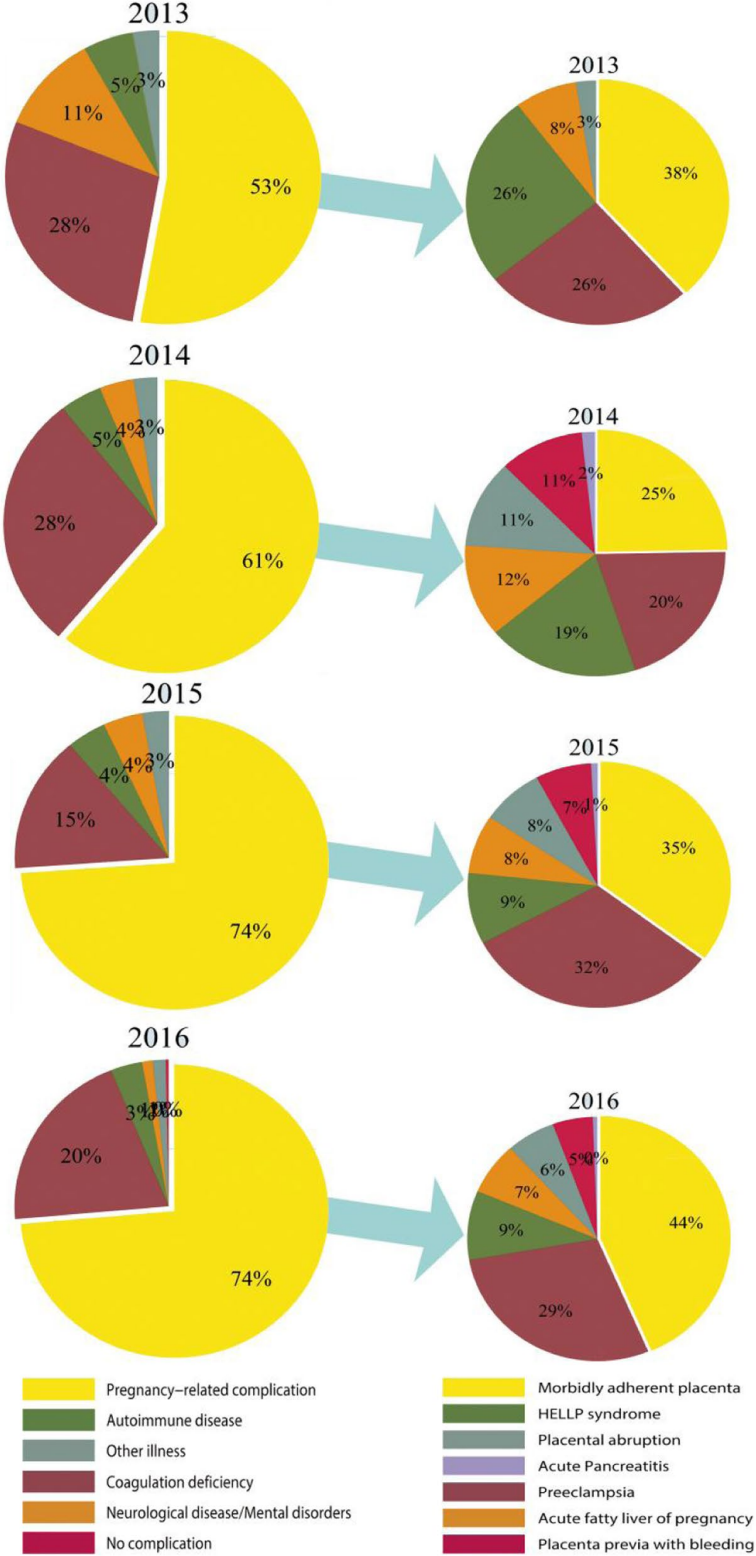
The chart of maternal complications and pregnancy-related complications yearly from 2013 to 2016

Maternal complications were divided into five groups and a variety of subgroups in our study. For data analysis, we coded maternal complications into zero to five to represent a specific disease. Pregnancy-related complications were the largest set of obstetric complications, including morbidly adherent placenta (MAP), gestational hypertension, preeclampsia, HELLP syndrome, placenta previa with bleeding, placental abruption, acute fatty liver of pregnancy, acute pancreatitis and gestational diabetes.

The chart showed that pregnancy-related complications, coagulation deficiency and autoimmune disease with abnormal coagulation were the three main reasons for choosing general anesthesia rather than neuraxial anesthesia during 2013–2016, accounting for 92%-97%. Further analysis showed that MAP, preeclampsia and HELLP syndrome were consistently the top 3 maternal complications among a variety of pregnancy-related complications. Overall, the occurrence of MAP increased from 38% in 2013 to 44% in 2016.

### Chart of total maternal complications and pregnancy-related complications in the GA group during 2013–2016 (Fig. 4)

**Fig. 4:**
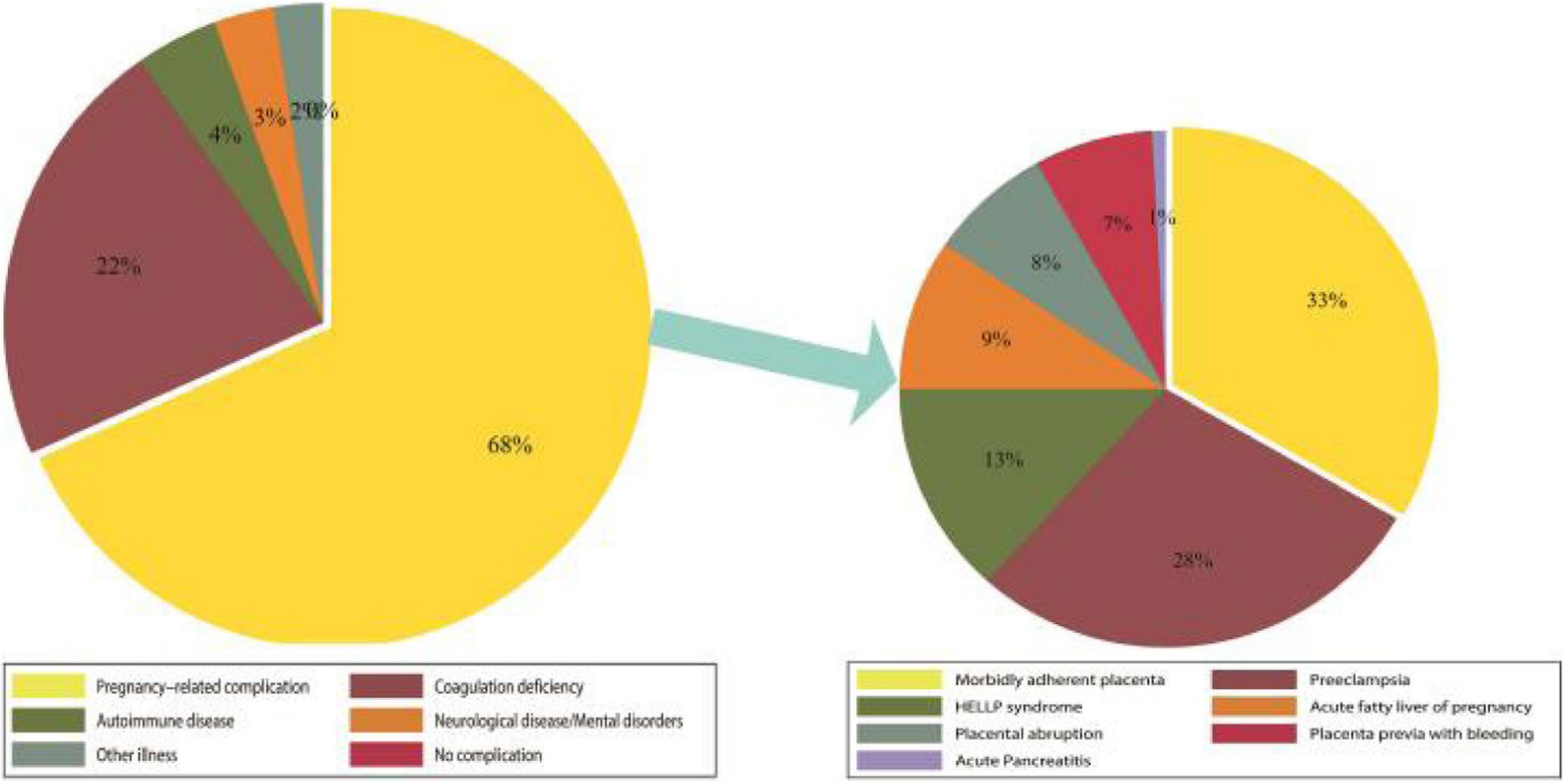
The chart of total maternal complications and pregnancy-related complications during 2013–2016

In total, during 2013–2016, pregnancy-related complications accounted for 68% of cases that chose general anesthesia, and coagulation deficiency was the second rank, accounting for 22%. Corresponding to the above, MAP was the first reason for general anesthesia among pregnancy-related complications, which accounted for 33%, preeclampsia ranked second, accounting for 28%, and HELLP syndrome accounted for 13%.

### Maternal characteristics and obstetric complications and newborn outcomes of parturients accepted cesarean delivery with neuraxial anesthesia and general anesthesia during 2013–2016 (Table 2)

**Table 2:** Maternal characteristics and obstetric complications and newborn outcomes of parturients who underwent CD with NA and GA during 2013–2016

Variables	NA (*N* = 1825)	GA (*N* = 761)	*P* value^#^
**Maternal Age (years)**	30.52 ± 4.95	30.25 ± 5.21	0.212
**Stratified Age (years)**			
18–28	678(37.15)	299(39.29)	0.538
29–33	641(35.12)	259(34.03)	
34–49	506(27.73)	200(26.28)	
Missing	0(0)	3(0.39)	
**Delivery Year**			
2013	188(10.3)	74(9.72)	0.931
2014	460(25.21)	200(26.28)	
2015	420(23.01)	173(22.73)	
2016	757(41.48)	314(41.26)	
**BMI (Kg/m** ^**2**^ **)**	28.88 ± 4.31	28.54 ± 4.27	0.188
**Stratified BMI (Kg/m** ^**2**^ **)**			
< 25.5	300(16.44)	129(16.95)	0.612
25.5–28	509(27.89)	203(26.68)	
28–31	478(26.19)	183(24.05)	
> = 31	473(25.92)	170(22.34)	
Missing	65(3.56)	76(9.99)	
**Gestational age (Days)**	266.28 ± 17.04	249.30 ± 24.03	< 0.001
**Stratified Gestational age (Days)**			
175–259	419(22.96)	449(59.00)	< 0.001
260–270	501(27.45)	182(23.92)	
271–277	488(26.74)	76(9.99)	
278–301	416(22.79)	53(6.96)	
Missing	1(0.05)	1(0.13)	
**Categorization of Maternal complications**			
No	1207(66.14)	1(0.13)	< 0.001
Nuerological disease and/or mental disorders	7(0.38)	24(3.15)	
Coagulation deficiency	2(0.11)	168(22.08)	
Pregnancy-related Complications	374(20.49)	520(68.33)	
Autoimmune disease	18(0.99)	30(3.94)	
Others	211(11.56)	18(2.37)	
Missing	6(0.33)	0(0)	
**Emergency or Selective**			
Emergency	941(51.56)	558(73.32)	< 0.001
Selective	883(48.38)	185(24.31)	
Missing	1(0.05)	18(2.37)	
**Primary or Repeat**			
Primary	1246(68.27)	421(55.32)	< 0.001
Repeat	574(31.45)	329(43.23)	
Missing	5(0.27)	11(1.45)	
**Twins**			
No	1729(94.74)	727(95.53)	0.401
Yes	96(5.26)	34(4.47)	
**Newborn weight(g)**	3260.8 ± 702.2	2652.4 ± 821.3	< 0.001
**Apgar Score at 1 min**			
0–4	11(0.6)	49(6.44)	< 0.001
5–7	31(1.7)	80(10.51)	
8–10	1782(97.64)	606(79.63)	
Death	1(0.05)	26(3.42)	
**Apgar Score at 5 min**			
0–4	7(0.38)	15(1.97)	< 0.001
5–7	12(0.66)	42(5.52)	
8–10	1805(98.9)	678(89.09)	
Death	1(0.05)	26(3.42)	
**NICU**			
0	1512(82.85)	497(65.31)	< 0.001
1	310(16.99)	232(30.49)	
Missing	3(0.16)	32(4.2)	
**Total Hospital Days**	7.34 ± 5.45	11.93 ± 9.63	< 0.001
**Hospital Days after Delivery**	4.39 ± 1.77	6.49 ± 4.20	< 0.001

*Indications: GA* General anesthesia, *NA* Neuraxial anesthesia, *BMI* Body mass index, *NICU* Neonatal intensive care unit

^#^*p* values were derived using chis-squared test or Fisher exact test for each categorical variable and Wilcoxon rank sum test for continuous variables, after excluding the corresponding missing value

Differences are supposed to exist in maternal complications and newborn outcomes between the GA group and the NA group. To evaluate these differences, we also collected electronic records of cesarean delivery cases who accepted neuraxial anesthesia during 2013–2016. To ensure the feasibility and efficiency of records in the NA group, we extracted some cases from the NA group according to maternal age and delivery year matched with the GA group at a ratio of 3:1. Owing to variable records missing in the NA group, 1825 cases with NA were enrolled.

No differences in maternal age or maternal BMI at delivery were observed between the GA and NA groups. Maternal complications and gestational age were significantly different between the GA and NA groups (*p* < 0.001). The rate of repeat cesarean delivery was higher in the GA group than in the NA group (*p* value < 0.001). The total hospital days and hospital days after delivery in the GA group were longer than those in the NA group (*p* value < 0.001). There were significant differences in newborn outcomes between the GA and NA groups, including newborn weight, newborn Apgar score at 1 min and 5 min and newborn admission to the NICU. Further data analysis showed that newborn mean gestation age (249.30 days versus 266.28 days) and newborn mean weight (2652.4 g versus 3260.8 g) presented great differences between the GA and NA groups, *p* < 0.001. The Apgar at 1 min and 5 min in the GA group were lower than those in the NA group. Newborns admitted to the neonatal ICU accounted for 30.49% in the GA group, most of them owing to gestational age < 34 weeks, and preterm infants (gestational age < 37 weeks) accounted for 59% in the GA group.

### Logistic regression for risk factors for a newborn Apgar score < 8 at 1 min or 5 min (Table 3)

**Table 3:** Logistic regression for risk factors for a newborn Apgar score < 8 at 1 min or 5 min

**Variables**	**Crude** **OR (%CI)**	**Adjusted** **OR (%CI)** ^a^
**Maternal complications**		
0/1	1.00(reference)	1.00(reference)
2	3.598(1.349 ~ 9.593)	0.999(0.336 ~ 2.969)
3	20.509(11.553 ~ 36.407)	4.476(2.228 ~ 8.993)
4	7.11(1.952 ~ 25.892)	2.109(0.52 ~ 8.561)
5	5.963(2.728 ~ 13.036)	5.282(2.324 ~ 12.007)
**Anesthesia Way**		
Neuraxial Anesthesia	1.00(reference)	1.00(reference)
General Anesthesia	9.482(6.723 ~ 13.373)	3.479(2.247 ~ 5.387)
**Emergency or Selective**		
Selective	1.00(reference)	1.00(reference)
Emergency	3.469(2.358 ~ 5.106)	1.989(1.31 ~ 3.021)
**Stratified Gestational age (Days)**		
175–259	24.308(8.95 ~ 66.019)	8.227(2.922 ~ 23.168)
260–270	1.502(0.46 ~ 4.907)	0.952(0.286 ~ 3.176)
271–277	2.929(0.958 ~ 8.961)	2.763(0.882 ~ 8.655)
278–301	1.00(reference)	1.00(reference)
P for trend	< 0.001	0.002

Indications: 0 represented normal without obstetric complications; 1 represented neurological disease and/or mental disorders; 2 represented coagulation deficiency; 3 represented pregnancy-related complications; 4 represented autoimmune disease with abnormal coagulation; 5 represented other limited complications

^a^ The model contained all variables in this table

Univariate logistic regression and multiple logistic regression showed that gestational age below 259 days (37 weeks) was the most risk factor for a newborn Apgar score < 8 (OR = 24.308 and 8.227, respectively). Pregnancy-related complications and other severe complications were also risk factors for a newborn Apgar score < 8, with high OR values (OR = 20.509 and 4.476, respectively). General anesthesia was another risk factor for a low Apgar score (OR = 9.482 and 3.479, respectively). Risk factors interacted with each other. At all events, maternal complications were associated with preterm delivery and choice of general anesthesia, and the newborn Apgar score was lower in the GA group.

## Discussion

There is consensus that neuraxial anesthesia (NA) is currently the optimized technique for cesarean delivery (CD), while general anesthesia (GA) is less often chosen than NA. This practice has been associated with decreased maternal adverse outcomes, especially maternal airway/respiratory complications, which accounted for 21% of claims related to anesthesia before 1990 in the United States [8, 9].

General anesthesia for cesarean delivery has been considered at risk for maternal difficult intubation and neonatal respiratory complications compared with NA [10, 11]. Although neuraxial anesthesia is predominant for cesarean delivery in our hospital, the rise of general anesthesia cannot be ignored. In our hospital, GA accounted for 3.17% of cesarean deliveries in 2013 and 10.23% in 2016, and the trend of GA increased significantly (*p* < 0.01) during 2013–2016 (Fig. 1). As data showed that multiple causes led to choosing GA for CD, pregnancy-related complications, especially MAP, were the largest maternal complication (Fig. 3). In total, MAP accounted for 33% of pregnancy- related complications in the GA group during 2013–2016 (Fig. 4).

MAP is clearly associated with severe postpartum hemorrhage (PPH), massive blood transfusion, and even death [12, 13]. In India, maternal mortality in patients with MAP accounts for 30% [14]. In our hospital, MAP was the major cause for massive blood transfusion, accounting for 55% of massive blood transfusions (RBC transfusions > 10 U) during 2013–2016 [15]. Accordingly, the incidence of MAP is associated with repeat CD and the number of earlier CDs [16, 17]. In China, population-based data of primary and repeat CD are still absent [18]. In our study, repeat CD accounted for 16.22% in the GA group in 2013, however, increased to 54.14% in the GA group in 2016, with a great growing number and increasing proportion during 2013–2016 (Table 1).

A multidisciplinary team (MDT), including obstetricians, anesthesiologists, radiologists, interventional service, and pediatricians, has worked together perioperatively to reduce the volume of blood loss and decrease the choose of general anesthesia among parturients with MAP in the latest two years.

With the advance of technology and airway devices, fewer difficult airway for CD during 2013–2016 were recorded in our hospital. From the end of 2013, we began to try using laryngeal mask airway (LMA) for CD with the capacity of gastric decompression. In 2013, LMA accounted for 28.38% (21/74), while LMA accounted for 92.99% (292/314) in 2016 with a rapid increase (Fig. 2). Generally, LMA was suggested for use as a rescue method after failing tracheal intubation for CD [19, 20]. Changes for using LMA to manage airways for laparoscopic operations with Trendelenburg position have been used since 2010. Accordingly, our practioners’ use of LMA forCD have increased cautiously with the rise of GA in OB unit. Per our institutional policy, LMA could be used with caution in cesarean delivery airway management if the parturient has been fasting for 6 h or longer. In addition, a gastric catheter will be placed into the stomach and connected with negative suction to empty the stomach. No clinical incidence of aspiration occurred in our clinical practice.

LMA is more tolerable and less irritating than tracheal intubation, and a lower dose of general anesthetics is needed in anesthesia induction compared with ETT. Success with LMA in airway management in our hospital may benefit from parturients with less morbid obesity (average BMI at delivery < 30 kg/m^2^) (Table 1), and another reason was the objective estimation of gastric volume with ultrasound.

Abnormal coagulation is the second risk factor for GA in CD procedures (Figs. 3 and 4). This group was mainly comprised of low platelet count and minor coagulation deficiency with prolonged coagulation time and/or low fibrinogen. There is still no consensus on the safe limit of platelet count for NA technique [21]. In our hospital, once the platelet count is below 80,000/dl, NA should be avoided; if the platelet count is between 80,000/dl ~ 100,000/dl, NA should be used with caution. In the latest two years, to decrease GA, single spinal anesthesia with 25G needle is administered to parturients whose platelet count between 75,000/dl and 100,000/dl.

Preeclampsia and HELLP syndrome were classified into pregnancy-related complications with coagulopathy in our study, which were of noteworthy reasons to choose GA inferior to MAP (Figs. 3 and 4). The trend of preeclampsia and HELLP syndrome was reported to be increasing in Taiwan and in the U.S. [22, 23]. The incidence of coagulopathy in patients with HELLP syndrome accounts for 30% [24]. A rapidly decreasing PLT count is another characteristic feature of HELLP syndrome [25]. Commonly in our hospital, blood routine test is required to re-examine 6–12 h before CD to determine anesthesia method according to platelet count.

Systemic lupus erythematosus (SLE) and antiphospholipid syndrome (APS) were the two main autoimmune diseases involved in our study, and APS partly coexisted with SLE (Figs. 3 and 4). Parturients with SLE and APS are associated with a higher risk of obstetric complications, including preeclampsia, hypertension, low platelet count, postpartum stroke, and even death [26, 27]. Recently, low-dose aspirin (LDA) and low molecular weight heparin (LMWH) have been suggested for parturients with SLE and/or APS to reduce high risk of venous thromboembolism and high risk of obstetric complications during pregnancy [28]. If the time interval is less than the longest duration suggested, for instance, time interval below 12 h in prophylactic dose and 24 h in therapeutic dose, we prefer choosing GA to avoid potential hematoma complications.

Other maternal complications, including individual preference, umbilical cord prolapse, severe heart failure, acute severe respiratory failure and acute severe pancreatitis, were classified into other groups owing to limited numbers.

To observe newborn outcomes, we collected additional newborn data from CD cases with NA. Our data showed that the newborn Apgar scores at 1 min and 5 min in the GA group were lower than those in the NA group, and the rate of newborn NICU admission was higher in the GA group than in the NA group (*p* < 0.001) (Table 2). Further data analysis showed that the newborn mean gestation age of 249.30 days and newborn mean weight of 2652.4 g in the GA group were lower than those of 266.28 days and 3260.8 g in the NA group.

Logistic regression was further used to identify risk factors associated with a newborn Apgar score below eight (Table 3). The results showed that gestational age < 259 days (equal to 37 weeks) was the strongest correlation factor, pregnancy related complications was another risk factor ranked second, and general anesthesia was the third related factor to a newborn Apgar score below eight. Even after adjustment, these three factors were still the most important risk factors. Clearly, interactions among these three factors cannot be distinguished from each other. For instance, low gestational age was in response to preterm delivery, maternal complications were the root cause for preterm delivery, and GA was associated with maternal complications. At all events, the newborn Apgar score was lower in the GA group, and GA caused by maternal complications was a risk factor for an Apgar score below eight beyond negligence.

Anesthesiologists need to be alert to newborn depression from general anesthetics crossing the placental barrier in the first 5 min after cord clamping. It is reasonable for us to suggest that newborns be monitored in a neonatal incubator for at least 5 min in the operating room, and an additional 5 min should be better. Tactile stimulation is suggested to support newborn breathing and mask oxygenation as necessary. Certainly, we need not worry about newborn overdepression by anesthetics at clinical dosages.

Reducing general anesthesia administration for cesarean delivery in our study mostly depends on lowering the incidence of massive hemmorrhage and related maternal and neonatal adverse outcomes associated with MAP. It is supposed that controlling nonclinical primary cesarean delivery is an effective method to reduce repeat cesarean delivery and MAP complications [5].

Fortunately, data analysis in China showed that the rate of primary CD has decreased [29]. The trend of GA for CD may be altered in the coming years, accompanied by changes in maternal characteristics and progress in lowering obstetric complications.

The limitation of our study is the lack of data on blood loss between the GA group and NA group, and we could not estimate the effect of general anesthesia on blood loss. Another limitation is that our data focus on a tertiary hospital, and the hospital is a transmitted center of parturients with severe maternal complications; thus, there may be a selective bias to population-based data. With BIS monitoring to direct enough depth of anesthesia, few claims for awareness in cesarean delivery procedures have been reported. Regretfully, no accurate electronic medical records of awareness can be collected.

## Data Availability

The datasets used and/or analyzed during the current study available from the corresponding author on reasonable request.
